# 351. Epidemiology and antifungal susceptibilities of clinically significant filamentous fungi from a tertiary hospital in Singapore from 2018 – 2021

**DOI:** 10.1093/ofid/ofac492.429

**Published:** 2022-12-15

**Authors:** Wenjie Huang, Mei Gie Tan, Yen Ee Tan

**Affiliations:** Singapore General Hospital, Singapore, Singapore; Singapore General Hospital, Singapore, Singapore; Singapore General Hospital, Singapore, Singapore

## Abstract

**Background:**

Filamentous fungi infections are associated with significant morbidity. Although an increase in antifungal resistance has been reported globally, no data pertaining to Singapore is available. This study investigates the species distribution and antifungal susceptibility profiles of molds isolated from a tertiary hospital in Singapore.

**Methods:**

A retrospective review of fungal cultures with antifungal susceptibility results reported in the laboratory information system from 2018 to 2021 was conducted. Unique isolates per patient were included. Molds were identified primarily by morphology. Molecular studies were used as an adjunct where required. Susceptibility testing was performed via broth microdilution (Sensititre YeastOne) and gradient diffusion (Liofilchem) methods.

**Results:**

125 isolates were analyzed (Table 1). The most common molds recovered were *Aspergillus*, *Fusarium* and *Mucorales*. *Aspergillus* and *Mucorales* frequently involved the respiratory tract and skin and soft tissue (SSTI) whereas *Fusarium* was isolated from SSTI and blood.

Amphotericin B had moderate activity against all molds, with only 11 out of 88 isolates (12.5%) being non-wild type (Table 2). The echinocandins exhibited good activity against *Aspergillus* and other hyaline molds but not *Fusarium* and *Mucorales*. The triazoles were most useful against *Aspergillus*, with posaconazole demonstrating the lowest geometric mean of 0.067. *Fusarium* had high MICs when tested against the azoles. Posaconazole was the azole of choice for *Mucorales*.

Six *Aspergillus* isolates (all *A. fumigatus*) were non-wild type when tested against voriconazole, with a MIC >1. Of these six isolates, three were also non-wild type for amphotericin B, with a MIC >2. In particular, one *Aspergillus fumigatus* complex isolate demonstrated resistance across all tested azoles and sequencing revealed Cyp51 mutations.

**Figure:**
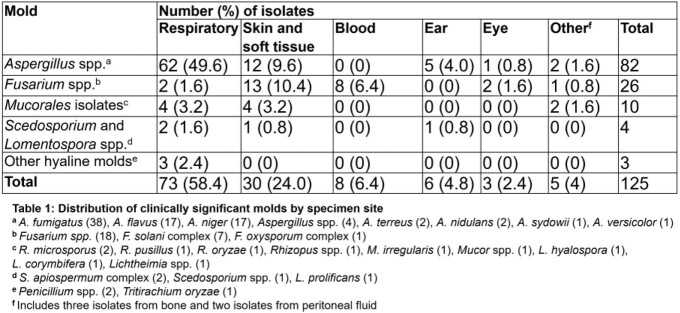

**Figure:**
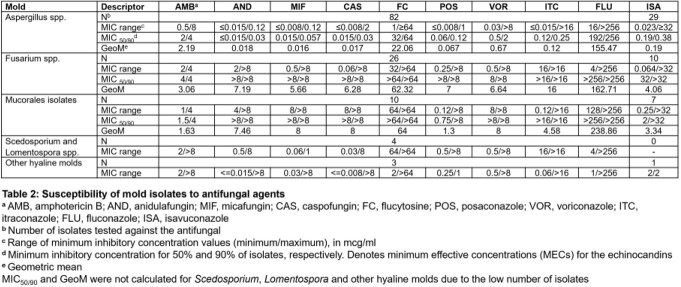

**Conclusion:**

*Aspergillus* species is the most prevalent clinically significant mold in our hospital. Although triazoles and echinocandins exhibit good activity, 13.33% of *Aspergillus* isolates were non-wild type for amphotericin B. Given the detection of these non-wild type *Aspergillus* species, susceptibility testing may be indicated in seriously ill patients to aid clinicians in selecting antifungal therapy.

**Disclosures:**

**All Authors**: No reported disclosures.

